# Phytochemical Evaluation of *Terminalia catappa* L. Extracts with Antibacterial and Antibiotic Potentiation Activities Against β-Lactam Drug-Resistant Bacteria

**DOI:** 10.3390/ijms27010177

**Published:** 2025-12-23

**Authors:** Muhammad Jawad Zai, Matthew James Cheesman, Ian Edwin Cock

**Affiliations:** 1 School of Environment and Science, Griffith University, Brisbane, QLD 4111, Australia; muhammadjawad.zai@griffithuni.edu.au; 2 Centre for Planetary Health and Food Security, Griffith University, Brisbane, QLD 4111, Australia; 3 School of Pharmacy and Medical Sciences, Griffith University, Southport, QLD 4222, Australia; m.cheesman@griffith.edu.au

**Keywords:** *Terminalia catappa*, antibiotic-resistance, MRSA, ESBL, flavonoid, tannin

## Abstract

*Terminalia catappa* L. (Family: Combretaceae) is used globally to treat various diseases, including bacterial infections. Whilst the antibacterial activity of *T. catappa* has previously been tested against antibiotic-sensitive bacterial strains, the antimicrobial activity against methicillin and β-lactam-resistant pathogens has been relatively ignored. The antibacterial activity of *T. catappa* extracts, both alone and combined with selected clinical antibiotics, was evaluated in this study. The inhibition of bacterial growth by the extracts was determined using agar diffusion and broth micro-dilution assays. Combinations of the extracts and several clinical antibiotics were also examined and the ∑FICs were calculated to determine the interaction class. Synergistic combinations were further evaluated by isobologram analysis. The *T. catappa* leaf extracts were screened for toxicity using *Artemia franciscana* lethality bioassays (ALA). Orbitrap liquid chromatography–mass spectrometry (LC-MS) profiling analysis was undertaken to identify flavonoid components of the extracts, putatively. The *T. catappa* methanolic extract inhibited all the tested bacterial strains. It displayed especially good inhibitory activity against *E. coli* (MIC = 130 µg/mL). Combining the *T. catappa* extracts with some conventional antibiotics potentiated the inhibitory activity of the combinations compared to the activity of individual components. LC-MS profiling analysis identified multiple flavonoid components, including rutin, quercitin, orientin, the tannin component, and ellagic acid in the extracts. All extracts were non-toxic against *Artemia* nauplii. The phytochemical constituents present in the *T. catappa* leaf extracts warrant future investigation as potential antibacterial agents.

## 1. Introduction

*Terminalia catappa* L. (family: Combretaceae) is a large tree found globally across tropical regions in coastal habitats. The leaves of *T. catappa* possess antioxidant and anti-inflammatory activities, as well as having antibacterial properties [1,2]. *Terminalia catappa* is listed in the Pharmacopeia Vegetables of the Caribbean and its leaves are used as a decoction to treat conditions such as urinary infections and gastritis [3]. In Suriname, *T. catappa* is used against gastrointestinal pathogens causing dysentery and diarrhea [4]. In Indian, Philippino, and Malaysian traditional medicine, *T. catappa* is also used to treat diarrhea [5,6]. Additionally, a plaster of *T. catappa* is used to treat skin infections in India [7]. Notably, many of these conditions result from bacterial infections, with *Escherichia coli* and *Staphylococcus aureus* being particularly frequent causes of diarrhea [8] and skin infections [9], respectively.

Due to its ethnobotanical uses, several studies have screened *T. catappa* extracts against selected bacterial pathogens [10]. An aqueous *T. catappa* leaf extract was recently reported to inhibit the growth of both Gram-positive (*Bacillus* spp. and *Staphylococcus* spp.) and Gram-negative bacterial pathogens (*E. coli*, *Klebsiella* spp., *Pseudomonas* spp.) [11]. However, that study did not quantify antimicrobial potency by calculating the minimum inhibitory concentrations (MICs) for the extracts. Instead, the authors only tested the activity of the aqueous extract at a single volume, without mentioning the concentration. Other studies also reported inhibition of gastrointestinal bacteria for an ethanolic extract obtained from the bark of this plant [12]. Similarly, an ethanolic *T. catappa* extract inhibited the growth of a panel of pathogens [13]. Against some bacterial strains, the *T. catappa* extracts showed moderate to good antimicrobial activity when compared to the standard antibiotic ciprofloxacin (10 µg/mL) [14]. *Terminalia catappa* bark also possesses antibacterial activity, with ethyl acetate, water, and hexane bark extracts inhibiting *Escherichia coli*, *Vibrio cholera*, *Pseudomonas aeruginosa* and *Klebsiella pneumoniae* growth in disc diffusion assays [15]. The greatest inhibitory activity in terms of zone of inhibition (ZOI) was recorded against *K. pneumoniae* (29 mm) and *E. coli* (26 mm) [15]. Unfortunately, that study did not determine MIC values, making it difficult to compare the potency between studies. Further studies quantifying the strength of the *T. catappa* extracts as antibacterial treatments are required to allow for comparisons between studies.

Additionally, aqueous *T. catappa* leaf extracts inhibit the growth of *Bacillus cereus*, *Shigella dysenteriae*, *S*. *aureus* and *Salmonella typhi* [14]. However, the findings of that study are dubious, as the MIC of the *T. catappa* extract was reported to be ~100 mg/mL against all pathogens. However, these MIC values are generally considered to be very high and would usually be classified as lacking antibacterial activity [15]. Whilst the authors describe these as MIC values, it is possible that the authors are reporting the lower of the doses they tested, rather than an MIC. This may account for the very high values reported in that study, although this should be verified. In contrast, our study tested a range of substantially lower concentrations and quantified the MIC, thereby allowing the potency of the extracts to be compared with other studies. Additionally, many of the earlier studies only examined the activity of aqueous or ethanolic extracts (but not both), whilst our study also investigated the antibacterial activity of extracts prepared using varying polarity solvents (methanol, water and ethyl acetate).

Notably, none of the earlier studies examined the antibacterial activity of *T. catappa* leaf extracts against methicillin-resistant bacterial pathogens or extended-spectrum β-lactamase (ESBL)-producing strains. As antibiotic resistance is increasingly common in strains of these bacteria, therapies that inhibit multidrug-resistant (MDR) strains are preferable for new antibiotic therapies. The present study assesses the antibacterial properties of *T. catappa* extracts on a range of bacterial species that are considered antibiotic-sensitive strains, as well as against corresponding MDR strains. Specifically, this study screened against antibiotic-sensitive *E. coli* and *K. pneumoniae* bacterial strains, as well as their β-lactam-resistant ESBL counterparts. Additionally, a β-lactam-sensitive *S. aureus* strain and an MRSA strain were also screened. Whilst our study focused on β-lactam resistance, previous studies have also reported that these strains are resistant to several other classes of antibiotics [16]. The emergence of β-lactam-resistant bacterial strains is particularly concerning, given our dependence on this antibiotic class to treat a broad range of infections. These pathogens cause substantial numbers of fatalities, and many strains have developed resistance to multiple antibiotics. The growth inhibition exerted by plant extracts was also determined in combination with tetracycline, chloramphenicol, ciprofloxacin, gentamicin, and erythromycin to investigate whether the *T. catappa* extracts potentiate their inhibitory activity. Additionally, metabolomic liquid chromatography–mass spectrometry (LC-MS) profiling of the *T. catappa* leaf extracts was used to identify and highlight noteworthy phytochemicals in the leaf extracts. *Artemia franciscana* (brine shrimp) lethality assays (ALAs) were used to test the toxicity of the *T. catappa* leaf extracts.

## 2. Results

### 2.1. Bacterial Susceptibility Screening

**Figure 1:**
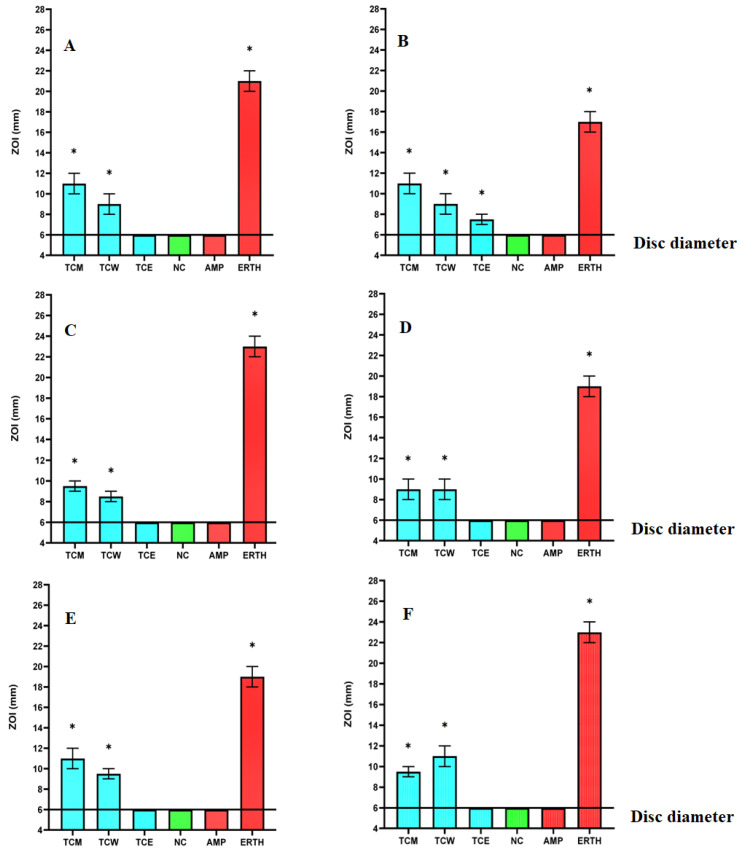
Disc diffusion susceptibility evaluation of the *T. catappa* leaf extracts against selected β-lactam sensitive and resistant bacterial panels: (**A**) *E. coli*; (**B**) ESBL *E. coli*; (**C**) *S. aureus*; (**D**) MRSA; (**E**) *K. pneumoniae*; (**F**) ESBL *K. pneumoniae*. TCM = *Terminalia catappa* methanol extract; TCW = *Terminalia catappa* water extract; TCE = *Terminalia catappa* ethyl acetate extract. Positive controls include AMP (ampicillin, 2 μg) and ERTH (erythromycin, 10 μg). Sterile deionised water was included as a negative control (NC). Zones of inhibition (ZOIs) are expressed as the means ± SEM of three independent experiments, each with internal triplicates (*n* = 9). One-way ANOVA was used to compare the results and values significantly different from the negative control (*p* < 0.01) are indicated with an asterisk (*). The disc diameter is indicated by the line at 6 mm on the *y*-axis.

Dried and powdered *T. catappa* leaves were mixed with a panel of mid to high polarity solvents and allowed to extract. Following filtration, the extracted material was allowed to air dry and weighed to calculate the masses and extraction yields of the extracts. The *T. catappa* leaf extracts were then resuspended individually in 10 mL of sterile deionised water (containing 1% DMSO). The concentrations of the methanol, aqueous, and ethyl acetate extracts were determined to be 8.3, 13.3 and 2 mg/mL, respectively. The antibiotic resistance profile of each bacterial strain was evaluated using β-lactam antibiotics. Additionally, the susceptibility of the bacteria to selected antibiotics from several other classes was also tested. Disc diffusion susceptibility assays (recorded as zones of inhibition; ZOIs) were used to approximate solid surface infections (Figure 1). Liquid microdilution assays were performed in 96-well plates, and MICs were calculated (Table 1). Notably, the antibacterial resistance/susceptibility trends were generally consistent between the solid-phase and liquid microdilution assays. The methanolic *T. catappa* methanol (TCM) and aqueous *T. catappa* (TCW) extracts each displayed growth inhibitory activity against all of the bacterial strains (both β-lactam sensitive and resistant strains) screened in this study. In contrast, the *T. catappa* ethyl acetate leaf extract (TCE) was completely ineffective in the liquid microdilution assay, although it inhibited ESBL *E. coli* growth in the solid-phase diffusion assays. The methanolic extract inhibited the growth of all of the bacterial strains tested. It was especially effective against the antibiotic-sensitive *E. coli* strain (MIC = 130 µg/mL). Notably, the methanolic extract (MIC = 259 µg/mL) was a more potent inhibitor of ESBL *K. pneumoniae* growth than it was against the susceptible *K. pneumoniae* strain (519 µg/mL). The aqueous extract demonstrated similar activity, albeit with lower efficacy against both *S. aureus* and MRSA (1663 µg/mL).

### 2.2. Quantification of Fractional Inhibitory Concentration (FIC)

Combinations of *T. catappa* extracts with the selected conventional antibiotics resulted in multiple interaction effects against the panel of β-lactam-sensitive and resistant strains (Table 2). The combination of TCW and tetracycline, as well as the TCM and chloramphenicol combination, induced synergistic effects against *S. aureus* and *K. pneumonia,* respectively. Additionally, seven combinations were additive, another twenty-two combinations were non-interactive, whilst three combinations were determined to be antagonistic.

### 2.3. Isobologram Analysis of Synergistic Combinations

**Figure 2:**
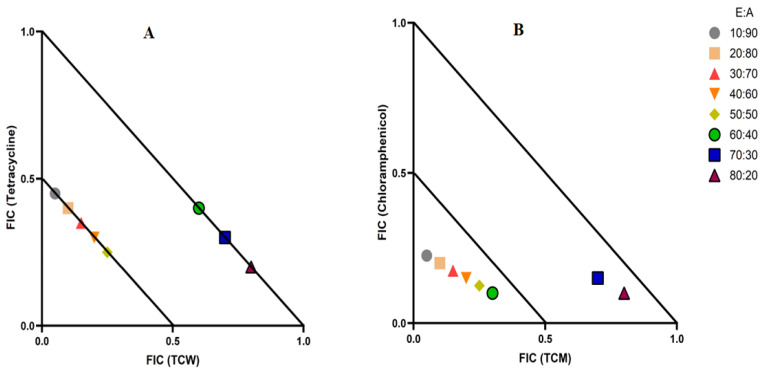
(**A**) Isobologram of the TCW extract in combination with tetracycline, when tested at various ratios against *S. aureus*. (**B**) Isobologram of the TCM extract in combination with chloramphenicol, when tested at various ratios against *K. pneumoniae*. Results represent the mean MIC values of two repeats. E:A ratio = % extract: % antibiotic. The ≤0.5/0.5 line represents synergy; the segment between the >0.5/0.5–≤1/1 lines represents additive interactions. Only synergistic and additive ratios are displayed in this graph.

Interestingly, the combination of TCW and tetracycline, as well as the combination of TCM and chloramphenicol, produced synergistic effects (Table 2). Therefore, these combinations were selected for further screening at various extract: antibiotic ratios by isobologram analysis to identify the synergistic ratios. In Figure 2, only the synergistic and additive ratios are depicted. As the non-interactive and the antagonistic combinations were outside the scale, they were excluded from this figure. The TCW and tetracycline combination only produced synergistic growth inhibitory effects when tested against *S. aureus* at ratios that contained 10–50% extract, whilst combinations containing 60–80% extract were classified as additive. Notably, the additive ratios have increased effects compared to the use of either component as an *S. aureus* monotherapy, although they would be less effective than using the combination at ratios that induce synergy. The TCM extract and chloramphenicol combination produced a synergistic interaction at ratios containing 10–60% extract, whilst combinations containing 70–80% extract produced additive effects. In contrast, the combination containing 90% TCM and 10% chloramphenicol was non-interactive, signifying that this ratio had no additional benefits over the use of the individual components.

### 2.4. HPLC-MS Identification of TCM and TCW Extract Compounds

**Figure 3:**
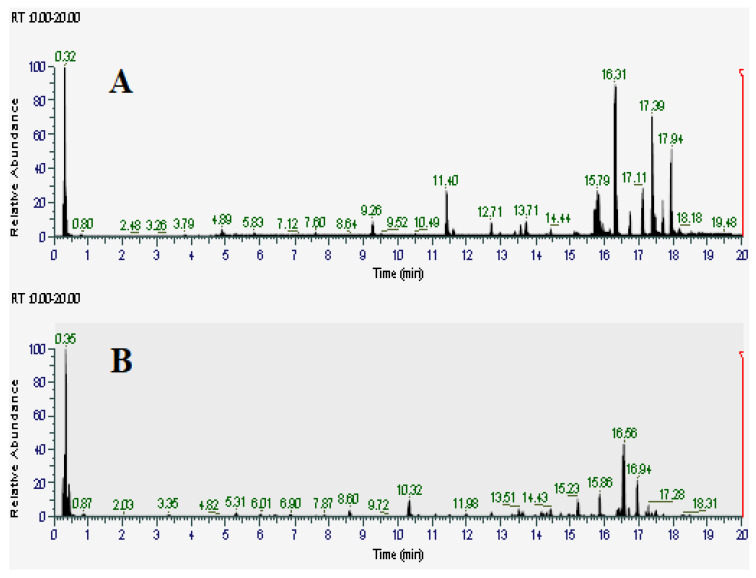
Positive ion LC-MS total compound chromatograms of (**A**) TCM (*Terminalia catappa* methanol) and (**B**) TCW (*Terminalia catappa* water) extracts.

Since the TCM and TCW extracts showed the greatest inhibitory activity, they were deemed to be the most promising and therefore were further analyzed for their phytochemical composition. Metabolomic fingerprints of those extracts were obtained using HPLC-MS, with a focus on the tannin and flavonoid constituents. Figure 3A,B show the total compound chromatograms (TCC) of the TCM and TCW extracts in positive ionization mode, respectively. The majority of compounds eluted isocratically early in the chromatogram at 95% water, indicating that the majority of compounds in both extracts are relatively high polarity. Compound Discoverer software (version 3.3; Thermo Scientific, Waltham, MA, USA) was used to putatively identify phytochemical components by comparing their mass spectral properties and retention indexes with the Natural Product Unknown ID and Local Database Searches databases. The empirical formula of each mass signal was determined based on the isotope abundance ratio using Xcalibur software (version 4.5; Thermo Scientific, Waltham, MA, USA). Deviations between the *m*/*z* values of the mass spectrum and the reported *m*/*z* values were limited to 5 ppm. Compounds with *m*/*z* ratios > 5 ppm compared to reported values were considered to be low confidence identities and were excluded from this study. Fragmentation patterns obtained from the primary and secondary mass spectra were subsequently cross-referenced with multiple databases (*m*/*z* Cloud, ChemSpider and Masslist). Only compounds that completely matched at least one of these databases were selected. Additionally, a literature search was conducted to further confirm the identity of the individual compounds. Several noteworthy flavonoid and tannin components were identified (Table 3).

### 2.5. Evaluation of Toxicity

Standardized ALA bioassays were employed to evaluate the apparent toxicity of the *T. catappa* leaf extracts. The extracts were tested across a 125 to 1000 µg/mL concentration range, and the results were graphed. Probit analysis was used to calculate the concentration of the extracts that caused mortality in 50% of the nauplii and defined as LC50 values. Notably, as the LC50 values of all of the extracts were determined to be >1000 μg/mL (results not presented), all of the *T. catappa* extracts were classified as nontoxic. In contrast, exposure to 2 mg/mL of potassium dichromate (positive control) caused death in 100% of the nauplii, indicating that the assay was functioning correctly. In contrast, exposure to seawater (negative control) induced 0% mortality following 24 h exposure.

## 3. Discussion

The *T. catappa* leaf methanol and water extracts both inhibited the growth of all the different bacterial strains tested herein (including the antibiotic-resistant strains). The growth inhibitory activity of the methanolic leaf extract was determined to be the most potent, with the lowest MIC values determined. In contrast, the ethyl acetate *T. catappa* leaf extract only inhibited ESBL *K. pneumoniae* growth in the agar diffusion assay, whilst it was ineffective in the liquid microdilution assay. These apparent differences may relate to the extraction yields for the solvents, and/or to the phytochemical compounds extracted using the distinct solvents. Lower polarity solvents generally only extract lower polarity compounds, and in lower relative abundances than higher polarity solvents [17]. Indeed, methanol and water extract a broad spectrum of phytochemical classes (particularly polar constituents), including flavonoids and tannins. In contrast, ethyl acetate has medium polarity and extracts substantially fewer phytochemicals (and in lower abundances) than the higher polarity extracts. The variations in the presence and relative abundance of the phytochemical components between the extracts may result in substantial differences in the apparent antibacterial potency observed between the solid-phase disc diffusion assays and the liquid microdilution assays. The diffusion of large and/or low polarity phytochemicals is retarded in solid-phase agar media, thereby influencing evaluation of the apparent antimicrobial potency [17]. Moreover, volatile compounds may evaporate from the gel surface, thereby diminishing the concentration diffusing through the gel, consequently affecting the evaluation of their apparent effectiveness [18]. The polarity of the extract components also influences their apparent efficacy in disc diffusion assays, as polar compounds diffuse more rapidly than lower polarity molecules, resulting in underestimated MIC values [19]. In comparison, liquid microdilution assays have greater sensitivity and are less influenced by molecular size and polarity.

The *T. catappa* extracts examined in this study exhibited good growth inhibitory activity against an antibiotic-sensitive Gram-negative gastrointestinal *E. coli* (MIC = 130 µg/mL) and against the corresponding ESBL antibiotic-resistant *E. coli* strain (MIC = 259 µg/mL; Table 1). Previous studies have also reported noteworthy antibacterial activity for a *T. catappa* extract prepared by similar methods against an antibiotic-sensitive strain of *E. coli*, although that study did not screen the extracts against antibiotic-resistant strains, nor was the potency of the extracts quantified by determining MIC values [20]. Instead, the inhibitory activity of the *T. catappa* extracts was only examined in that study using disc diffusion assays (ZOI = 14 ± 0.58 mm), which were similar to the ZOI values measured in our study (11 ± 0.50 mm). Another study extracted 50 g of dried *T.catappa* leaves in 200 mL of hydroalcohol (9:1 of methanol/water) and tested its growth inhibitory activity against a single *E. coli* strain [21]. The extract was only tested against the bacterium at a 2000 µg/mL concentration, and no inhibitory activity was detected in that study. In contrast, in our study, we extracted one gram of plant material in various solvents of different polarities and tested the resultant extracts to quantify the MIC. The inhibition of *E. coli* by *T. catappa* leaf extracts in our study (and in several earlier studies) validates the traditional use of this plant to treat gastrointestinal ailments. Future studies to test the growth inhibitory activity of *T. catappa* leaf extracts against other gastrointestinal pathogens, including *Shigella* and *Salmonella* species, are required, and studies are planned in our group to test the extracts against an extended panel of gastrointestinal pathogens.

The *T. catappa* extracts tested in our study displayed good activity in liquid microdilution assays when screened against the antibiotic-sensitive *S. aureus* and MRSA strains (MIC = 259 µg/mL against both) (Table 1). Other studies have assessed the activity of *T. catappa* extracts against *S. aureus* using only disc diffusion assays (ZOI 14.5 ± 0.28 mm) [20], which is higher than the ZOIs measured in our study (9 ± 0.25 mm). Notably, the activity reported in those studies was only evaluated against an antibiotic-sensitive strain of *S. aureus.* In contrast, our study also screened the *T. catappa* extracts against a methicillin-resistant *S. aureus* strain. Other previous studies have also reported growth inhibitory activity for *T. catappa* leaf extracts prepared in a similar manner against *S. aureus* when tested at a high dose of 2000 µg/mL [21]. Unfortunately, that study did not quantify the potency of the extract by calculating MIC values, and it is therefore difficult to comment on the potency of the extracts or to compare with other studies. The inhibition of *S. aureus* and MRSA by the *T. catappa* extracts in our study (and the earlier studies) validates its traditional use to treat skin diseases in several traditional healing systems.

It is possible that the *T. catappa* leaf extract constituents may exert their antibacterial effects through alternative mechanisms to those of the clinical antibiotics (particularly the β-lactam class of antibiotics) towards which these bacteria are resistant. Conversely, these plant extracts may contain compounds capable of mitigating bacterial antibiotic resistance. This finding is encouraging as the bacterial strains examined herein also show significantly reduced susceptibilities to multiple classes of antibiotics, including aminoglycosides, fluoroquinolones, macrolides, sulfonamides and tetracyclines, as well as β-lactams [16]. Additionally, the *T. catappa* leaf extracts inhibited the growth of both the Gram-positive and Gram-negative bacteria tested in this study, thereby highlighting their potential as targets for the development of novel broad-spectrum antibiotic therapies. To more extensively evaluate their antibacterial potential, future studies should test the *T. catappa* extracts against an extended panel of bacterial species, including multidrug-resistant (MDR) strains and evaluate their mode of action, including the inhibition of biofilm.

Multiple recent reports have shown promising trends for combining plant extracts with conventional antibiotics to synergise their antimicrobial efficacy against a range of bacterial strains [22]. Our study identified two synergistic, seven additives, twenty-two non-interactive and three antagonistic interactions. Antimicrobial effectiveness is substantially enhanced more for synergistic interactions compared to additive interactions. Therefore, whilst additive interactions are an improvement compared to the use of either component as a monotherapy, synergistic interactions offer substantially greater benefit. Our study observed synergistic interactions for combinations containing TCW and tetracycline against *S. aureus*, and also for the TCM extract and chloramphenicol combination against the *K. pneumoniae* strains tested. The TCM, TCW and TCEs were nontoxic when tested in the ALA toxicity bioassay, thereby indicating their safety for use in combination therapies. In contrast, antagonistic interactions diminish the efficacy of the extract and antibiotic components in combinations and should therefore be avoided.

**Figure 4:**
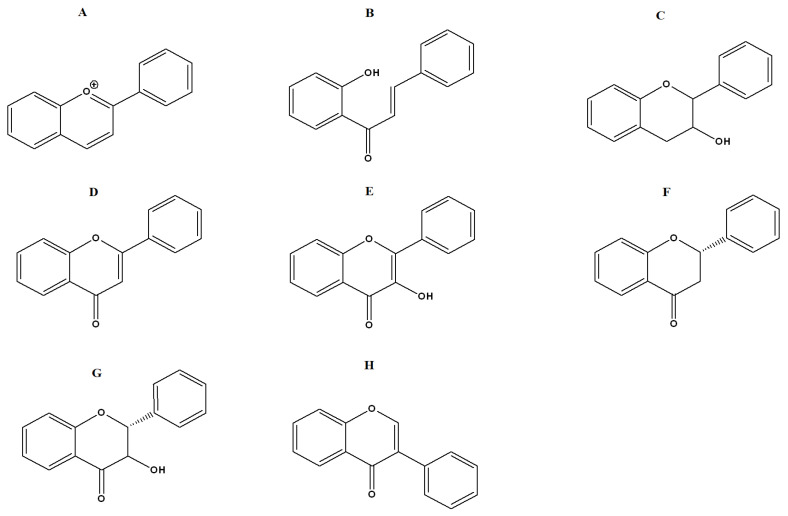
Classes of flavonoids. (**A**) anthocyanidins; (**B**) chalcone; (**C**) flavan-3-ol; (**D**) flavone; (**E**) flavonol; (**F**) flavanone; (**G**) flavononol; and (**H**) isoflavone.

**Figure 5:**
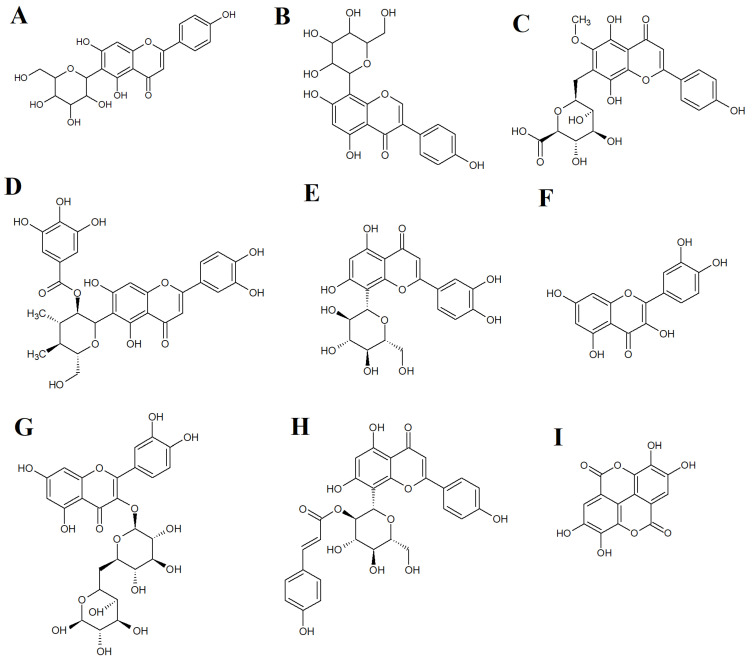
(**A**) 1,5-anhydro-1-[5,7-dihydroxy-2-(4-hydroxyphenyl)-4-oxo-4H-chromen-6-yl] hexitol; (**B**) 1,5-anhydro-1-[5,7-dihydroxy-3-(4-hydroxyphenyl)-4-oxo-4H-chromen-8-yl] hexitol; (**C**) hispidulin 7-glucuronide; (**D**) isoorientin 2″-O-gallate; (**E**) orientin; (**F**) quercetin; (**G**) rutin; (**H**) vitexin 2″-O-*p*-coumarate; and (**I**) ellagic acid.

At least thirty-nine *Terminalia* spp. have previously undergone phytochemical studies, resulting in the identification of 368 compounds [23]. These compounds include flavonoids, tannins, terpenoids, phenylpropanoids, simple phenolics and other chemical constituents. Our study focused on flavonoids and tannins as several compounds; these phytochemical classes are generally known to have antibacterial properties against multiple pathogenic bacteria [24,25]. Flavonoids are classified as anthocyanidins (Figure 4A), chalcones (Figure 4B), flavanols (Figure 4C), flavones (Figure 4D), flavonols (Figure 4E), flavanones (Figure 4F), flavononols (Figure 4G), and isoflavones (Figure 4H) based on their chemical structures. Tannins are generally categorized into hydrolysable or condensed tannins. Hydrolysable tannins can be hydrolyzed into their monomeric units in acidic or basic conditions or using enzymatic treatments. In contrast, condensed tannins are resistant to acid hydrolysis. In this study, several flavonoids and one tannin were identified in the *T. catappa* extracts. These included the flavonoids 1,5-anhydro-1-[5,7-dihydroxy-2-(4-hydroxyphenyl)-4-oxo-4*H*-chromen-6-yl] hexitol (Figure 5A), 1,5-anhydro-1-[5,7-dihydroxy-3-(4-hydroxyphenyl)-4-oxo-4*H*-chromen-8-yl] hexitol (Figure 5B), hispidulin 7-glucuronide (Figure 5C), isoorientin 2″-*O*-gallate (Figure 5D), orientin (Figure 5E), quercetin (Figure 5F), rutin (Figure 5G) and vitexin 2″-*O*-*p*-coumarate (Figure 5H). The tannin ellagic acid (Figure 5I) was also identified in our study. Previous studies have also identified a variety of flavonoids, including quercetin and several tannins, in extracts prepared using Indian-grown *T. catappa* extracts [26]. Additionally, *T. catappa* sourced from Ghana has also been shown to be rich in flavonoids and tannins [27].

Multiple studies have reported antibacterial effects for plant extracts that contain flavonoids and tannins against various bacterial pathogens. Notably, the growth inhibitory activity of kaempferol 3-*O*-*α*-l-(2‴,4‴-di-*E*-*p*-coumaroyl)-rhamnoside and kaempferol 3-*O*-*α*-l-(2″-*Z*-*p*-coumaroyl-4‴-*E*-*p*-coumaroyl)-rhamnoside was reported to be superior against MRSA compared to the standard antibiotics ciprofloxacin, erythromycin, norfloxacin, tetracycline, and oxacillin, with MIC values 0.5–2 µg/mL [28]. Structure-activity relationship studies for these compounds highlighted the importance of 5, 7-dihydroxylation of ring A and 4′-hydroxylation of the B ring for anti-MRSA activity [29]. The structure of flavonoids (particularly substitutions on the aromatic ring) also influences their antibacterial activity. As further plant extracts exhibiting antibacterial activities are discovered, it is likely that an expanding number of flavonoids will be identified as antibacterial agents. Flavonoids, including quercetin, which was also identified during LC-MS analysis in this study, are good antibiotic candidates, either for use alone or in combination with clinical antibiotics [30]. Studies have explored the potential mechanism of action of flavonols, with a focus on three specific β-ketoacyl carrier synthases as key targets for novel antibiotic design. For example, 3,6-dihydroxyflavone demonstrated antibacterial efficacy against an extremely drug-resistant *E. coli* strain by inhibiting β-ketoacyl carrier protein synthase I [31]. Notably, the flavonoid kaempferol-3-rutinoside (isolated from *Sophora japonica* L. flowers) substantially inhibits the growth of *Streptococcus mutans* via inhibition of sortase A enzymatic activity, which is crucial for host invasion and adhesion of Gram-positive bacteria [32].

Several tannins inhibit the growth of multiple microorganisms, including fungi, bacteria and yeasts [33]. Notably, most tannins have bacteriostatic effects, rather than bactericidal activity [34]. Biofilm formation is believed to be associated with a significant number of chronic bacterial infections, with greater than 60% of bacterial infections associated with the formation of biofilms [35]. Infections associated with biofilms pose specific challenges, as bacteria within biofilms exhibit increased resistance to the host immune system, as well as antibiotics and other biocides, compared to their planktonic counterparts. Interestingly, ellagic acid (which was putatively identified in our study in the *T. catappa* leaf extracts) inhibits the biofilm formation in selected *E. coli* strains by up to 26% [36]. Unlike traditional antibiotics that have biocidal activities, ellagic acid specifically inhibits biofilm formation, whilst having minimal impact on beneficial and planktonic bacteria. Such compounds might not be as prone as conventional antibiotics to bacterial resistance mechanisms, although this remains to be verified. Additionally, it is likely that they will have lower impacts on beneficial microflora.

Whilst the tannin and flavonoid components that were identified in the *T. catappa* leaf extracts in our study may possess noteworthy antibacterial properties, they may also (or instead) potentiate the antibacterial properties of other extract components or conventional antibiotics. Additional studies are needed to confirm and characterize their antibacterial and antibiotic-potentiation potential. These compounds should be evaluated as potential antibiotic therapies in their own right, as well as for their ability to potentiate the growth inhibitory properties of other antibiotic components. Interestingly, the *T. catappa* leaf extracts demonstrated efficacy against both antibiotic-resistant and antibiotic-sensitive bacteria. This is a desirable characteristic for the development of new antibiotic therapies. Comprehensive characterization of the identified flavonoids and tannins is essential. Rigorous evaluation of their antimicrobial properties (alone and in combination with other extract compounds and/or conventional antibiotics) is needed to evaluate their antimicrobial potency and to determine their specific antibacterial mechanism(s). Additionally, some of the mass signals noted in the LC-MS chromatograms remain unidentified, and additional phytochemical investigation is required. Future studies should also evaluate the use of other phytochemical techniques, like ^1^H and ^13^CNMR, to obtain a clear picture of phytochemicals present in these extracts. All the extracts examined in this study were non-toxic in the ALA toxicity bioassay. Therefore, these extracts may be safe for therapeutic use, although their toxicity should be further tested in an extended human cell line panel before they can be considered safe for medicinal use.

## 4. Materials and Methods

### 4.1. Materials

Analytical grade (AR) solvents were purchased from Ajax Fine-Chemicals Ltd. (Taren Point, Australia) and used for all extractions. Mueller–Hinton agar and broth (Oxoid Ltd., Thebarton, Australia) were prepared following the manufacturer’s instructions. All other chemicals and reagents were obtained from Sigma Aldrich (Clayton, Australia) unless stated otherwise.

### 4.2. Plant Collection and Extraction

Fresh *Terminalia catappa* leaves were collected with permission of the garden’s botanist from Brisbane Botanical Gardens (Mt Coot-tha, Australia) and are stored as voucher specimens at Griffith University (Voucher code: Tcat_BGIC_2021L). The leaves were washed in sterile deionised water and then dehydrated to a constant mass using a Sunbeam^®^ food desiccator and the dried leaves were then milled to produce a fine powder. Individual tubes were filled with one-gram masses of the leaf powder and extracted in parallel with 50 mL of either methanol, sterile deionized water, or ethyl acetate. The extracts were mixed thoroughly and stored at room temperature for 24 h, followed by vacuum drying at 60 °C. The masses of the dried leaf extracts were recorded to allow for the calculation of extract concentrations. A minimal volume (100 µL) of dimethyl sulfoxide (DMSO) was used to aid in solubilising the extracts, and the volume was then increased to a total of 10 mL with the addition of sterile deionized water. Particulate matter was then removed using 0.22 µm syringe-driven filters (Millipore Pty Ltd., North Ryde, Australia). The resultant extracts were then aliquoted and stored at 4 °C until further use.

### 4.3. Antibacterial Studies

Modified Kirby–Bauer agar disc-diffusion susceptibility assays were utilized as a preliminary evaluation of the susceptibility of the bacteria to the extracts and conventional antibiotics. The MIC of each extract, along with control antibiotics and their combinations, was quantified using standard checkerboard liquid micro-dilution assays [37]. This method, known for its sensitivity and widespread application, enables the determination of MIC values and facilitates comparisons between studies, as well as between extracts within the same study.

### 4.4. Bacterial Culture Conditions

Powdered Mueller–Hinton broth and agar were purchased from Oxoid Ltd. (Thebarton, Australia) and the media were prepared according to the manufacturer’s guidelines. Each test bacteria stock solution was streaked separately onto individual Mueller–Hinton agar plates to verify culture purity before testing and to allow for the isolation of individual colonies. The Mueller–Hinton agar plates were incubated at 37 °C for 24 h to allow for colony growth. A single pure bacterial colony was subsequently picked from the agar surface and used to inoculate fresh Mueller–Hinton broth. The inoculated media were then incubated at 37 °C until the log phase of bacterial growth was attained (as judged by optical density (OD) measurements at 600 nm every 30 min until a stable growth plateau was achieved). The log growth culture was then used to make stock cultures by separately inoculating fresh Mueller–Hinton broth with 100 μL volumes of the individual cultures and incubating at 37 °C for 24 h.

### 4.5. Concentration of Antibiotic and INT Solution

The initial starting concentration of the antibiotic used in the assay was 2.5 µg/mL, and serial dilution was performed to determine the lowest dose of antibiotic that could inhibit bacterial growth. After incubating the plate for 24 h at 37 °C, 40 µL of 0.4 mg/mL INT (iodonitrotetrazolium) solution was dispensed in each well of the plate, followed by a waiting period for colour development. A pink-red colour indicates the presence of live bacteria, while the lack of colour development indicates inhibition of bacterial growth.

### 4.6. Examination of Combinational Effects

The *T. catappa* leaf extracts were combined with the selected clinical antibiotics at 1:1 ratios to examine the interactions between the components (as described in Section 2.3). This evaluation was based on a colorimetric analysis, with the MIC determined as the lowest dose that inhibited INT colour development. A positive control (antibiotic) and a negative control (water) were included on all plates, with all screenings conducted in triplicate (*n* = 3).

The fractional inhibitory concentration (FIC) values were calculated using the following formulae:

FIC (E) = (MIC of *T. catappa* leaf extract in combination)/(MIC of *T. catappa* leaf extract independently)

FIC (A) = (MIC of antibiotic in combination)/(MIC of antibiotic independently)

ΣFIC = FIC (E) + FIC (A).

ΣFIC values ≤ 0.5 were categorized as synergistic; 0.5–≤1.0 were classed as additive; >1.0–≤4.0 were designated as indifferent/non-interactive, and ΣFIC values > 4.0 were considered antagonistic.

### 4.7. Isobologram Analysis of Synergistic Combinations

Combinations that produce synergistic interactions were further analyzed across ratios ranging from 100% antibiotic to 0% extract (in 10% increments), in combination with inverse percentages of clinical antibiotic. All ratios were tested in duplicate and mean values were determined and used to calculate fractional inhibitory concentration (FIC) values. Isobologram graphs were plotted and used to identify ratios that induce synergy.

### 4.8. LC-MS Analysis

Putative identification of phytochemical phytoconstituents in the extracts used non-targeted metabolomic profiling techniques previously developed in our laboratory [37].

### 4.9. Toxicity Studies

The apparent toxicity of each extract was examined using ALA bioassays following standardized methods [17].

### 4.10. Data Analysis and Statistical Methods

Statistical significance between the control and treatment groups was evaluated using one-way ANOVA. While statistical analysis did not apply to the 96-well microtiter liquid dilution assay, the reliability of MIC values was ensured by performing the assays three times on separate days, with two replicates per assay (*n* = 6). This method verified the reproducibility of all flavonoids, antibiotics and their combinations.

## 5. Conclusions

The growing need to develop novel therapies to treat bacterial illnesses and infections (particularly against antibiotic-resistant bacterial strains) has focused attention on natural products as drug candidates for further study. Our findings demonstrate that *T. catappa* leaf extracts exhibit significant inhibitory effects on the growth of multidrug-resistant bacteria, which is comparable to their efficacy on sensitive strains. This suggests the presence of distinctive antibacterial mechanisms within specific components of the plant extracts. The identified compounds in this study may contribute to these observed activities and, thus, warrant future investigation into their potential as antibacterial agents. Additionally, further examination of the capacity of these compounds to potentiate the antibacterial effects of common clinical antibiotics (or other phytochemicals) is required.

## Figures and Tables

**Table 1:** Liquid dilution assay MIC values (µg/mL) of the *T. catappa* leaf extracts and selected conventional antibiotics against selected bacterial pathogens.

Test (Extract or Antibiotic)	Bacterial Species and MIC (µg/mL)					
	*E. coli*	ESBL *E. coli*	*S. aureus*	MRSA	*K. pneumoniae*	ESBL *K. pneumoniae*
TCM	130	259	259	259	519	259
TCW	416	1663	1663	1663	416	438
TCE	-	-	-	-	-	-
Tetracycline	-	-	1.25	-	-	-
Chloramphenicol	-	-	0.31	-	1.25	1.25
Ciprofloxacin	2.5	-	0.62	2.5	2.5	1.25
Gentamicin	0.039	0.039	0.03	0.03	0.03	0.03
Erythromycin	-	-	1.25	-	2.5	-
Negative control	-	-	-	-	-	-

MIC values for TCM = *Terminalia catappa* methanol extract, TCW = *Terminalia catappa* water extract, TCE = *Terminalia catappa* ethyl acetate extract; - indicates no inhibition at any concentration tested. Mean MIC values of triplicate determinations are shown and are expressed in µg/mL.

**Table 2:** ∑FIC values for interactions between plant extracts and antibiotics.

Bacteria	Extract	Tetracycline	Chloramphenicol	Ciprofloxacin	Gentamicin	Erythromycin
*E. coli*	TCM	-	-	**4.25**	3.10	-
	TCW	-	-	2.25	1.42	
	TCE	-	-	-	-	-
*ESBL E. coli*	TCM	-	-	-	2.85	-
	TCW	-	-	-	**5.33**	-
	TCE	-	-	-	-	-
*S. aureus*	TCM	1.25	**1**	1.50	1.12	1.25
	TCW	**0.50**	1.25	3	**0.66**	**1**
	TCE	-	-	-	-	-
MRSA	TCM	-	-	2.25	**5.70**	-
	TCW	-	-	**0.75**	2.66	-
	TCE	-	-	-	-	-
*K. pneumoniae*	TCM	-	**0.37**	**0.62**	2.72	**0.62**
	TCW	-	2.5	2.25	2.84	1.12
	TCE	-	-	-	-	-
ESBL *K. pneumoniae*	TCM	-	1.25	1.25	2.85	-
	TCW	-	**0.66**	2.5	1.41	-
	TCE	-	-	-	-	-

∑FIC values of plant extracts in combination with conventional antibiotics against sensitive and resistant strains of *E. coli*, *S. aureus* and *K. pneumoniae*. TCM = *Terminalia catappa* methanol extract; TCW = *Terminalia catappa* water extract; TCE = *Terminalia catappa* ethyl acetate extract; **Synergy = ≤0.5**; **Additive = >0.5–1.0**; Indifferent = >1.0–≤4; **Antagonistic = >4.0**. FIC evaluations were performed in duplicate. - indicates no inhibition at any concentration tested.

**Table 3:** Qualitative analysis of LC-MS of TCM and TCW.

**Flavonoids**	**Retention** **Time (min)**	**Empirical** **Formula**	**Molecular** **Mass**	**Putative Identification**	**Relative Abundance (% Total Area)**	
					**TCM**	**TCW**
	8.13	C_30_H_26_O_12_	578.14	Vitexin 2″-*O*-*p*-coumarate	0.02	
	6.35	C_27_H_30_O_16_	610.15	Rutin	0.05	
	6.30	C_15_H_10_O_7_	302.04	Quercitin	0.02	
	5.54	C_21_H_20_O_11_	448.10	Orientin	0.03	
	6.19	C_21_H_20_O_10_	432.10	1,5-Anhydro-1-[5,7-dihydroxy-2-(4-hydroxyphenyl)-4-oxo-4*H*-chromen-6-yl] hexitol	0.55	
	6.94	C_28_H_24_O_15_	600.11	Isoorientin 2”-*O*-gallate		0.01
	13.22	C_22_H_20_O_12_	476.09	Hispidulin 7-glucuronide		0.02
	7.35	C_21_H_20_O_10_	432.10	1,5-Anhydro-1-[5,7-dihydroxy-3-(4-hydroxyphenyl)-4-oxo-4*H*-chromen-8-yl] hexitol		0.18
**Tannins**	6.35	C_14_H_6_O_8_	302.00	Ellagic acid (isomer 1)	1.09	
	7.46	C_14_H_6_O_8_	302.00	Ellagic acid (isomer 2)		0.48

## Data Availability

The original contributions presented in this study are included in the article. Further inquiries can be directed to the corresponding author.

## Abbreviations

The following abbreviations are used in this manuscript:

ALA	*Artemia franciscana* nauplii lethality assay
ANOVA	Analysis of variance
AR	Analytical-grade reagent
DMSO	Dimethyl sulfoxide
ESBL	Extended-spectrum β-lactamase
FIC	Fractional inhibitory concentration
∑FIC	Sum of FIC values
HPLC-MS	High performance liquid chromatography coupled with mass spectrometry
INT	Iodonitrotetrazolium
LC-MS	Liquid chromatography–mass spectrometry
LC_50_	50% lethal concentration
MIC	Minimum inhibitory concentration
MDR	Multidrug resistant
MRSA	Methicillin-resistant *Staphylococcus aureus*
*m*/*z*	Mass-to-charge ratio
SEM	Standard error of the mean
TCC	Total compound chromatograms
TCE	*Terminalia catappa* ethyl acetate extract
TCM	*Terminalia catappa* methanolic extract
TCW	*Terminalia catappa* water extract
ZOI	Zone of inhibition
